# Pancreatic Cancer Surgical Resection Margins: Molecular Assessment by Mass Spectrometry Imaging

**DOI:** 10.1371/journal.pmed.1002108

**Published:** 2016-08-30

**Authors:** Livia S. Eberlin, Katherine Margulis, Ivette Planell-Mendez, Richard N. Zare, Robert Tibshirani, Teri A. Longacre, Moe Jalali, Jeffrey A. Norton, George A. Poultsides

**Affiliations:** 1 Department of Chemistry, Stanford University, Stanford, California, United States of America; 2 Department of Biomedical Data Sciences, Stanford University, Stanford, California, United States of America; 3 Department of Statistics, Stanford University, Stanford, California, United States of America; 4 Department of Pathology, Stanford University, Stanford, California, United States of America; 5 Department of Surgery, Stanford University, Stanford, California, United States of America; Harvard Medical School, UNITED STATES

## Abstract

**Background:**

Surgical resection with microscopically negative margins remains the main curative option for pancreatic cancer; however, in practice intraoperative delineation of resection margins is challenging. Ambient mass spectrometry imaging has emerged as a powerful technique for chemical imaging and real-time diagnosis of tissue samples. We applied an approach combining desorption electrospray ionization mass spectrometry imaging (DESI-MSI) with the least absolute shrinkage and selection operator (Lasso) statistical method to diagnose pancreatic tissue sections and prospectively evaluate surgical resection margins from pancreatic cancer surgery.

**Methods and Findings:**

Our methodology was developed and tested using 63 banked pancreatic cancer samples and 65 samples (tumor and specimen margins) collected prospectively during 32 pancreatectomies from February 27, 2013, to January 16, 2015. In total, mass spectra for 254,235 individual pixels were evaluated. When cross-validation was employed in the training set of samples, 98.1% agreement with histopathology was obtained. Using an independent set of samples, 98.6% agreement was achieved. We used a statistical approach to evaluate 177,727 mass spectra from samples with complex, mixed histology, achieving an agreement of 81%. The developed method showed agreement with frozen section evaluation of specimen margins in 24 of 32 surgical cases prospectively evaluated. In the remaining eight patients, margins were found to be positive by DESI-MSI/Lasso, but negative by frozen section analysis. The median overall survival after resection was only 10 mo for these eight patients as opposed to 26 mo for patients with negative margins by both techniques. This observation suggests that our method (as opposed to the standard method to date) was able to detect tumor involvement at the margin in patients who developed early recurrence. Nonetheless, a larger cohort of samples is needed to validate the findings described in this study. Careful evaluation of the long-term benefits to patients of the use of DESI-MSI for surgical margin evaluation is also needed to determine its value in clinical practice.

**Conclusions:**

Our findings provide evidence that the molecular information obtained by DESI-MSI/Lasso from pancreatic tissue samples has the potential to transform the evaluation of surgical specimens. With further development, we believe the described methodology could be routinely used for intraoperative surgical margin assessment of pancreatic cancer.

## Abstract 2

Richard Zare and colleagues develop and test a method to evaluate surgical resection margins from pancreatic cancer surgery using mass spectrometric imaging.

## Author Summary

**Why Was This Study Done?:**

- Ambient ionization mass spectrometry imaging can provide accurate diagnostic information differentiating cancerous from noncancerous tissue samples and has been recently shown to be particularly powerful in helping pathologists and surgeons determine whether cancer reaches the edge (margin) of the resection specimen in real time during surgery.
- This study was performed to evaluate the feasibility and efficacy of this method during surgery for pancreatic cancer, one of the most lethal human cancers.

**What Did the Researchers Do and Find?:**

- Our methodology was developed and tested using 63 banked pancreatic cancer samples and 65 samples (tumor and specimen margins) collected prospectively during 32 pancreatectomies performed from 2013 to 2015.
- We found that desorption electrospray ionization mass spectrometry imaging (DESI-MSI) allows discrimination of normal pancreatic and pancreatic cancer tissue based on diagnostic metabolic signatures and has the potential to assist in surgical decision making by informing the surgeon whether the entire tumor has been removed or not.

**What Do These Findings Mean?:**

- These findings provide novel information on molecular markers of pancreatic cancer and showcase the value of this methodology as an adjunct to the current pathologic method (frozen section analysis) for determining the completeness of cancer surgery.
- The data reported in this study could be made available during actual surgery, and allow the surgeon to extend the boundaries of surgery to remove any residual tumor discovered by DESI-MSI at the margin.

## Introduction

Resection of pancreatic cancer is a complex and technically demanding surgical procedure due to the location of the pancreas adjacent to many critical organs and vascular structures, and the locally infiltrative nature of pancreatic adenocarcinoma. Surgical resection of pancreatic cancer remains the main curative option for this disease. It is well accepted that complete resection with negative surgical margins is associated with improved long-term survival [1–4]. This finding calls for careful evaluation of surgical resection margins intraoperatively. Positive margins, defined as the presence of tumor cells at the specimen edge and/or the resection bed, have been associated with increased local recurrence and decreased overall survival [1]. During surgical resection, depending on the location of the tumor (in the head or body/tail of the gland), up to five surgical margins are typically evaluated by an expert team of pathologists using histologic analysis of frozen sections. These margins include the pancreatic neck margin, the retroperitoneal/uncinate process margin, the vascular groove margin, the gastric/proximal duodenal margin, and the bile duct margin. However, in practice, the accuracy of intraoperative delineation of pancreatectomy margins can be variable and subjective, with false-negative results occurring in up to 20%–30% of pancreatic adenocarcinoma patients [5–7]. Consequently, the need exists for developing more accurate, more time-efficient, and less operator-dependent technologies to evaluate specimen margins during pancreatic cancer surgery [8]. We have recently described an approach combining desorption electrospray ionization mass spectrometry imaging (DESI-MSI) with the least absolute shrinkage and selection operator (Lasso) statistical method to classify tissue sections and evaluate surgical resection margins during gastric cancer surgery [9]. We describe herein the application of this methodology to the intraoperative evaluation of pancreatic cancer surgical margins.

In the last few years, ambient ionization mass spectrometry (MS) has become a powerful approach for tissue imaging and diagnosis [10–13]. In particular, DESI-MSI is the most extensively used ambient ionization MS technique for chemical imaging and diagnosis of tissue samples [13]. In DESI-MSI, a spray of charged droplets is directed toward a tissue sample, allowing chemicals to be dissolved at the sample surface, ionized by mechanisms similar to electrospray ionization, and transferred into a mass spectrometer, where the mass-to-charge ratios (*m/z*) of molecular ions and their abundances are measured [14]. By rastering the tissue sample underneath the DESI-MSI spray spot and collecting a mass spectrum at every point, it is possible to determine the distribution of numerous molecular species with a typical spatial resolution of 200 μm at an acquisition rate of 0.5 s/pixel. Besides DESI-MSI, other ambient ionization techniques including probe electrospray ionization [15], solid-probe-assisted nanoelectrospray ionization [16], touch spray [17], and rapid evaporative ionization MS [18] have been used for cancer tissue diagnosis and surgical margin evaluation [13]. Various human cancer tissues have been investigated by ambient ionization MS, including liver [19], breast [20,21], brain [22], kidney [23], prostate [24,25], bladder [26], gastric [25], colorectal [25], and ovarian [26] cancers. In this study, we tested the usefulness of DESI-MSI to classify pancreatic tissue as cancerous or benign and to evaluate surgical margins collected from pancreatic cancer surgery.

## Methods

### Ethics Statement

Banked tissue samples were obtained without identifiable or clinical information. Therefore, the Stanford institutional review board (IRB) determined that this portion of the research did not include human subject research and was exempt from full review. However, Stanford’s IRB ethical review committee determined that the portion of the study involving patients was human subject research, and thus IRB approval was obtained. Approval was also obtained from Stanford’s Cancer Institute Scientific Review Committee. Written informed consent was obtained for all patients recruited.

### Banked Human Malignant and Benign Pancreatic Tissues

Sixty-three frozen human tissue specimens including pancreatic ductal carcinoma and benign pancreatic tissue were obtained from the Stanford Tissue Procurement Facility under approved IRB protocol. Samples were stored in a −80°C freezer until sectioned (15 μm thick) using a Leica CM1950 cryostat (Leica Microsystems). No sample size determination was done for this study. After sectioning, the glass slides were stored in a −80°C freezer. Prior to mass spectrometry imaging, the glass slides were dried in a desiccator for approximately 15 min.

### Prospective Collection of Surgical Samples

Thirty-two pancreatic cancer patients scheduled to undergo pancreatectomy at Stanford University Hospital were preoperatively consented for our study. All patients gave written consent under IRB approval (protocol number 25655), approved by Stanford’s IRB committee (IRB 7). Inclusion criteria were that the patient was scheduled to undergo surgery for pancreatic cancer removal. No exclusion criteria were applied. During surgery, the neck and/or retroperitoneal/uncinate margins of the specimen were subjected to frozen section histopathologic evaluation, as is routinely performed independently of our research. In parallel to the process of frozen section, adjacent 5- and 15-μm thick tissue sections of each margin were obtained for DESI-MSI. For all but one case, a sample of the tumor was obtained in addition to the surgical margins. In total, 65 surgical tissue samples were evaluated by DESI-MSI during the period from February 27, 2013, to January 16, 2015.

### Analyses

The MS and histologic analyses reported were chosen during monthly meetings between the leading investigators or a subgroup of the investigators. The analysis was planned a priori and was based on a recent successful investigation that the authors had performed in gastric cancer. The statistical methods were optimized during the study based on the results obtained and the need for refinement of the methods.

### Mass Spectrometry Imaging

A 2-D DESI-MSI source (Prosolia) coupled to an LTQ XL mass spectrometer (Thermo Scientific) was used for tissue imaging. DESI-MSI was performed in the negative ion mode from *m/z* 90 to *m/z* 1,200. The spatial resolution of the imaging experiments was 200 μm. The histologically compatible solvent system, dimethylformamide:acetonitrile 1:1 (v/v), was used for analysis at a flow rate of 0.8 μl/min. The N_2_ pressure was set to 175 psi. After DESI-MSI, the same tissue section was subjected to H&E staining for histopathologic evaluation by expert pathologist T. A. L. in a blind manner. For ion identification, tandem MS analyses were performed.

DESI-MSI data were collected on entire tissue sections. After DESI-MSI, the slides were stained and evaluated by the pathologist. Regions including lymphocytes, inflammation, and necrosis were observed in part of the samples, but were not selected within the pixels analyzed by statistical analysis because the goal of this study was to identify and discriminate cancer from normal pancreatic tissue, and not to characterize these other histologic features. DESI-MSI is performed as a sequence of line scans, and, thus, regions of glass slide (no tissue sample) are also analyzed. Pixels corresponding to the non-tissue area were imaged but not included in the pixels selected.

### Histopathologic Evaluation

The same tissue sections analyzed by DESI-MSI were subjected afterward to standard H&E staining protocol. These sections were adjacent and serial to, but not the exact same, sections used during surgery for surgical margin assessment. Pathologic evaluation was performed using light microscopy. Regions of benign pancreatic glands, normal stroma, and pancreatic cancer were delineated on the glass slides. Tissues that contained regions of mixed histology were evaluated and given a percent composition for each cell/tissue type (benign glands, normal stroma or pancreatic cancer).

### Statistical Analysis

The 2-D raw data obtained by DESI-MSI were converted to text files and imported to the R package for statistical analysis. The images were plotted in R and manually segmented into regions of interest as determined by histopathologic evaluation. Intensities for a total of 13,320 *m/z* values were recorded in each spectrum. To reduce complexity and account for small differences in registration between spectra, these MS features were averaged in nonoverlapping bins of six *m/z* values to yield a total of 2,220 features per spectrum. We randomly divided the patients into one training set and two sets of test samples. Within the training set, we applied the Lasso method (multiclass logistic regression with L1 penalty) using the glmnet 2.0–2 package in the CRAN R language library [27].

The Lasso is a shrinkage and selection method for supervised learning. It minimizes the usual sum of squared errors (or negative log-likelihood) with a bound on the sum of the absolute values of the coefficients. As a result, it yields a “sparse” solution containing the most informative features for the prediction task, that is, models that involve only a subset of the variables/predictors [28]. As such, it has an advantage over methods such as support vector machines, which are not designed to yield sparse solutions. On the other hand, if interactions between features are important, methods such as random forests and boosting may yield better results. (We tried these approaches here, and they did not offer improvement over the Lasso method.)

In this application, the Lasso method yields a model with parsimonious sets of features for discriminating between pancreatic cancer, normal pancreatic glands, and normal pancreatic stromal tissue. A mathematical weight for each statistically informative feature is calculated by the Lasso depending on the importance that the mass spectral feature has in characterizing a certain class. Features that do not contribute to a class of the linear model receive a weight of zero and are disregarded. An ion whose peak height, or abundance, is important for characterizing a certain class is given a positive weight, whereas ions for which low abundance or their absence is important receive a negative weight. Because the features selected by the Lasso can occur at a valley or a shoulder of an actual peak in the mass spectrum, identification of the selected features was performed by characterizing the nearest mass peak to the statistically selected feature.

Classification was done on a pixel by pixel basis into one of three classes: (1) benign glands, (2) cancer, or (3) stroma. We employed 25-fold cross-validation (CV), leaving out one patient at a time, to select the Lasso tuning parameter and to assess the predictive accuracy within the training set. Then, the chosen model was applied to the first test set of 15 patients, from which samples were of clear diagnosis. We then applied this method and an improved approach using customized training sets [29] to an independent set of mixed samples (including banked and surgical samples).

Note that the prediction performance is of main interest when evaluating the performance of the statistical classifier. *p*-Values, which are commonly used to evaluate the statistical significance of defined results between groups, are not the central focus in classification approaches with a large number of features, as in our study, and thus are not applicable to our results. Survival probabilities were calculated based on the Kaplan-Meier method and compared using the log-rank test.

## Results

### Molecular Imaging and Characterization of Pancreatic Tissue

**Fig 1:**
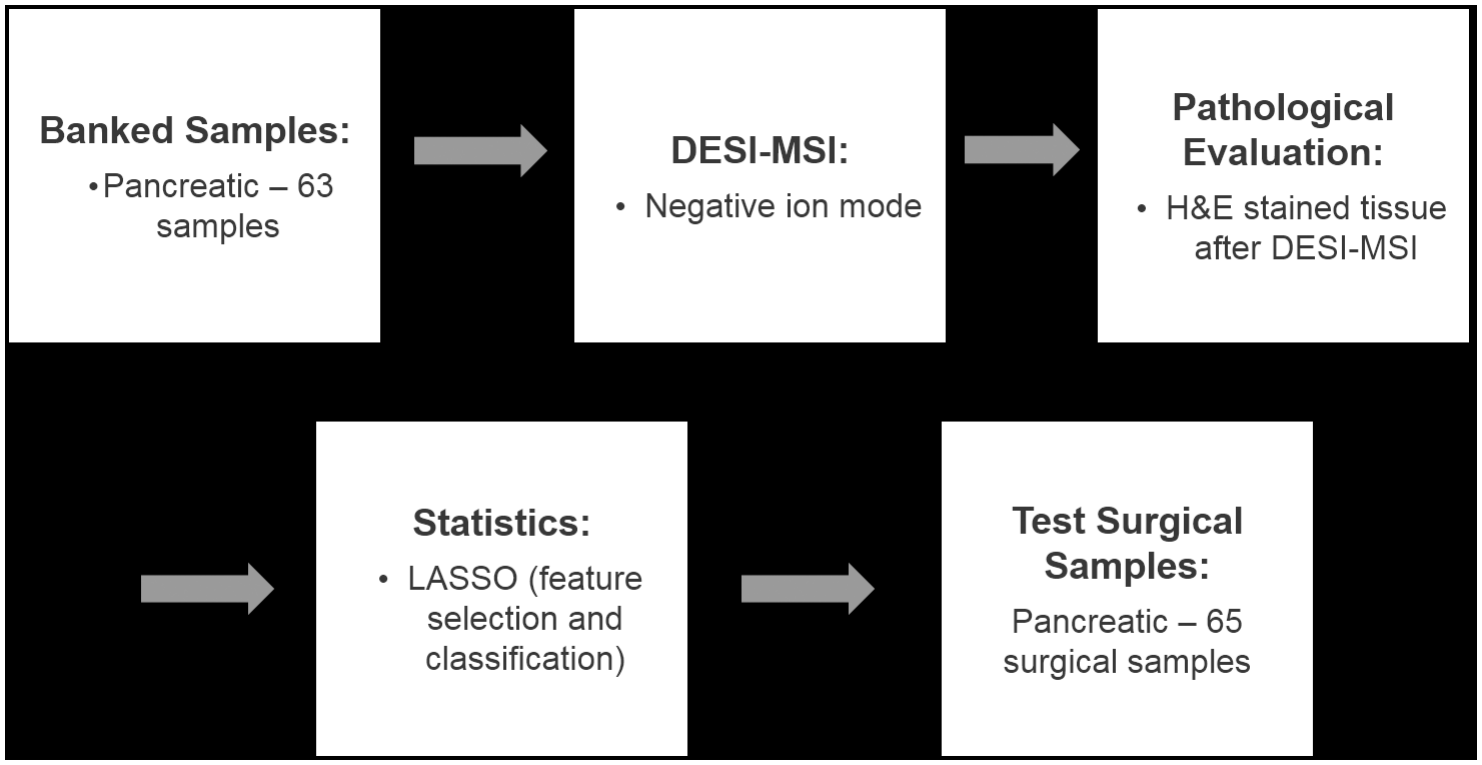
Flowchart summarizing the study performed.

A total of 128 human pancreatic tissues including banked specimens and tissue prospectively collected from surgeries for our study were analyzed in the negative ion mode by DESI-MSI. Fig 1 shows a flowchart of our study design. For the majority of the samples analyzed, the 2-D ion images obtained showed high heterogeneity in the distribution of molecular ions. Most of the heterogeneity in ion distribution was assigned by histopathologic evaluation as regions of cancer, normal pancreatic glands, or normal stromal tissue. Many samples presented highly mixed regions in which all of these histologic features were simultaneously observed. In a few samples, other histologic features were observed by the study pathologist including regions with acute inflammation, lymphocytes, and necrosis. These regions were not consistently seen across samples and were not considered in our approach owing to the lack of a statistically significant number of spectra.

**Fig 2:**
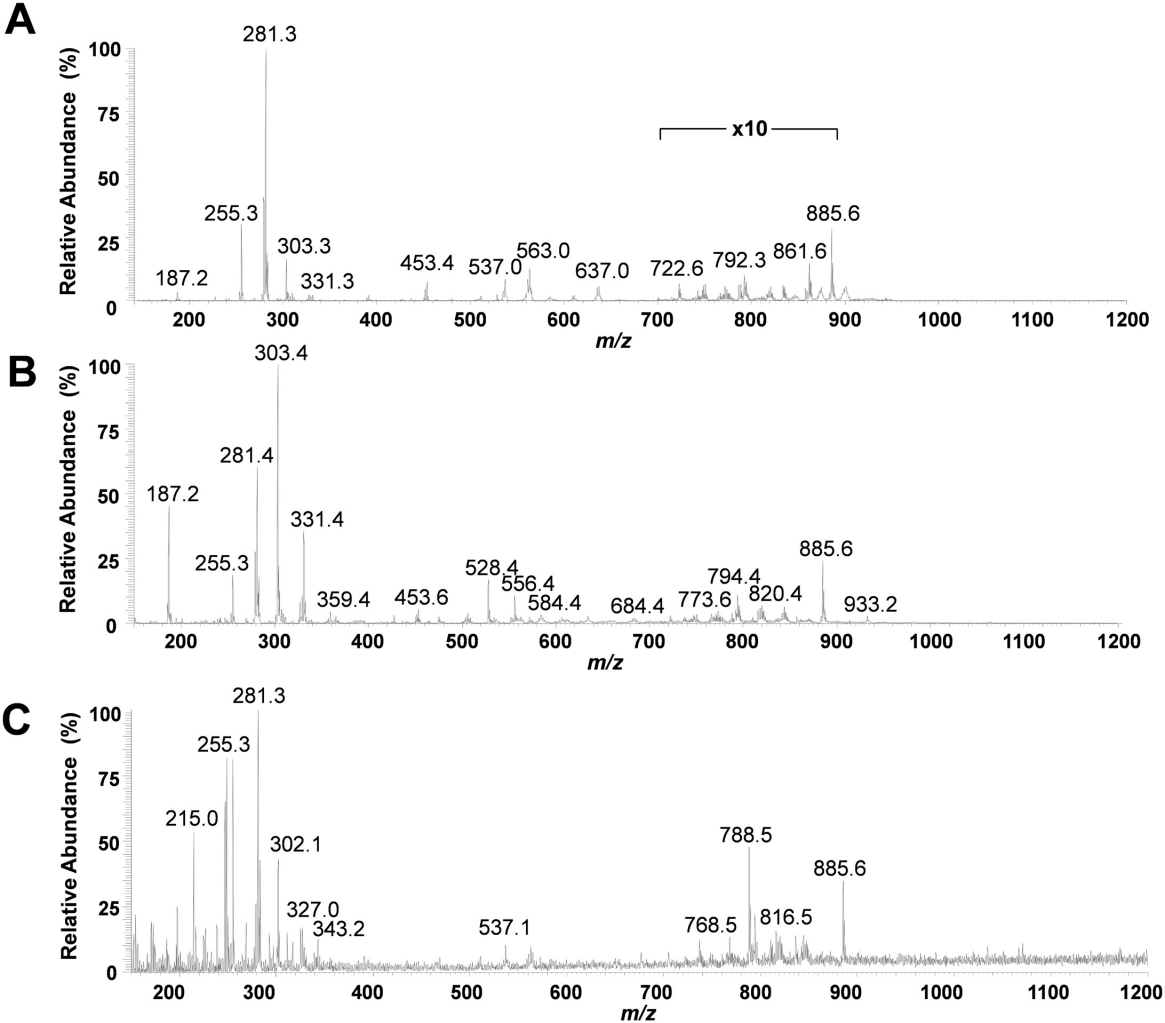
Representative negative ion mode DESI-MSI mass spectra obtained from pancreatic tissue. (A) Normal pancreatic glandular tissue from sample PC775 (note 10× zoom applied from *m/z* 700 to *m/z* 900 to assist visualization); (B) pancreatic cancer tissue from sample PC13702; and (C) normal pancreatic stromal tissue from sample PC0423.

**Fig 3:**
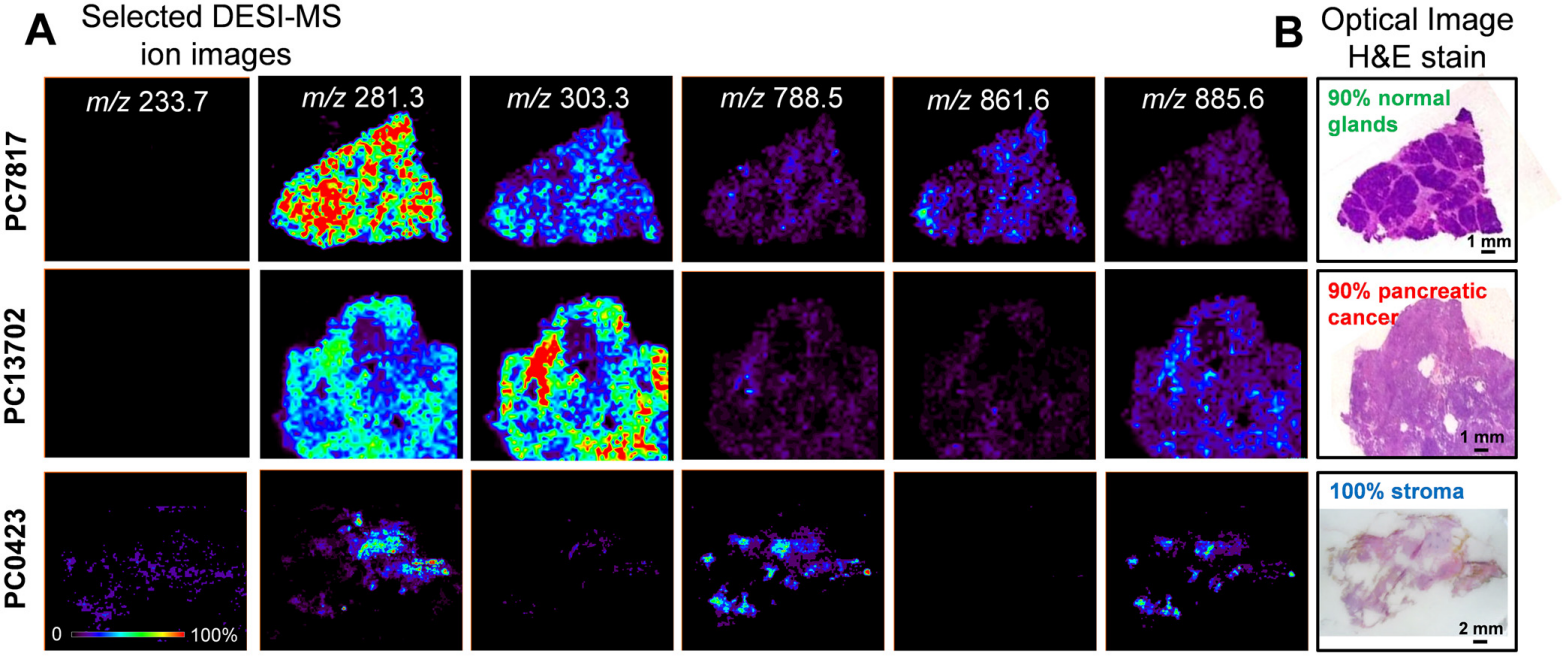
Selected 2-D negative ion mode DESI-MSI ion images obtained from pancreatic tissue samples. (A) Ion images of *m/z* 281.3 (oleic acid), *m/z* 303.3 (arachidonic acid), *m/z* 788.5 (PS(36:1)), *m/z* 861.6 (PI(36:2)), and *m/z* 885.6 (PI(38:4)) for samples PC7817, which is composed of 90% normal pancreatic glands and 10% normal stromal tissue, sample PC13702, which is composed of 90% pancreatic cancer and 10% normal stromal tissue, and sample PC0423, which is entirely composed of normal stroma. (B) Optical images of the same tissue sections subjected to H&E stain and evaluated by histopathology.

The mass spectra obtained for regions of normal pancreatic glands, normal stroma, and cancerous tissue presented high relative abundances of many molecular ions commonly attributed to lipid species in negative ion mode DESI-MSI mass spectra of human tissue (Fig 2). Normal pancreatic glands and cancerous regions showed high relative abundances of low *m/z* ions (*m/z* 200–400) attributed to free fatty acids (FAs), in comparison to higher *m/z* ions (*m/z* 700–1,000) attributed to glycerophospholipids (GPs). In particular, benign glands presented higher relative and total abundances of free FAs and FA dimers commonly observed in the *m/z* 500–600 range compared to both cancerous and stromal tissues. In normal pancreatic glands, the most abundant free FAs observed were identified as oleic acid (*m/z* 281.2), palmitic acid (*m/z* 255.3), and arachidonic acid (*m/z* 303.3). Dimers of these species were observed at *m/z* 537.0 (oleic and palmitic) and *m/z* 563.0 (oleic and oleic), among others. In the higher *m/z* range, GPs of various classes were observed including glycerophosphoinositol (PI) 38:4 at *m/z* 885.6, PI(36:2) at *m/z* 861.5, PI(34:2) at *m/z* 833.5, glycerophosphoglycerol (PG) 36:3 at *m/z* 771.5, and glycerophosphoetanolamine 37:5 at *m/z* 750.5. Cancerous tissue presented high relative abundances of polyunsaturated FAs including arachidonic acid (*m/z* 303.3) and adrenic (16-docosatetraenoic) acid (*m/z* 331.2). In the higher *m/z* range, chloride adducts of glycerophosphocholines (PCs) including PC(34:1) at *m/z* 794.4 and PC(34:0) at *m/z* 792.4, and other deprotonated GPs such as PG(36:2) at *m/z* 773.6 and PI(38:4) at *m/z* 885.6, were observed. Stromal tissue presented a characteristic lipid profile with an overall lower total lipid abundance (total mass spectrum ion counts) compared to normal pancreatic glands and cancerous tissue, and showed high relative intensities of oleic acid, palmitic acid, glycerophosphoserine (PS) 36:1 at *m/z* 788.8, PS(38:1) at *m/z* 816.5, and PI(38:4) at *m/z* 885.6. Fig 3 shows selected 2-D DESI-MSI ion images of the identified ions for the samples PC7817, which is composed of 90% normal pancreatic glands and 10% normal stromal tissue, PC13702, which is composed of 90% pancreatic cancer and 10% normal stromal tissue, and PC0423, which is composed of purely normal stroma.

### Statistical Approaches and Predictions

The large amount of molecular features obtained from the 254,235 pixels analyzed makes data interpretation difficult and calls for the use of sophisticated multivariate statistical techniques [9,30,31]. First, we implemented the approach we previously developed using the Lasso method to generate a statistical prediction based on DESI-MSI data [9,28]. To build our Lasso statistical classifier, a group of 42 samples with regions of clear histologic diagnosis (>90% composition of a single tissue type) was selected and randomly divided into a training set and a validation set of samples. Mixed samples with high cellular heterogeneity were evaluated separately as a test set of samples, and the results were individually analyzed for each sample. The training set of samples consisted of 25 samples with regions of pancreatic cancer, normal pancreatic glands, or normal stroma tissue, contributing a total of 45,273 spectra. Using the training set of samples, the Lasso selected a total of 112 *m/z* values that are important in characterizing all classes and that yielded the fewest CV errors (Table 1). From those, 59 different *m/z* values were selected by the classifier as important features to characterize pancreatic cancer, and 54 *m/z* values and 14 *m/z* values were selected as important features to characterize normal pancreatic glands and normal stroma, respectively. The statistical weight for each informative feature is calculated by the Lasso depending on the importance that the mass spectral feature has in characterizing a certain class. An ion whose relative abundance is important for characterizing a certain class is given a positive weight, whereas ions for which low relative abundance or their absence is important receive a negative weight. In this way, certain *m/z* values were selected as important for more than one class, with different statistical weights. For example, *m/z* 233.7 was selected as important for characterizing both normal glands and stroma tissues, with statistical weights of −0.146 and +0.983, respectively. On the other hand, *m/z* 738.8 was selected as important for characterizing pancreatic cancer and normal glands, with statistical weights of +0.130 and −0.148, respectively.

**Table 1:** *m/z* values selected by the Lasso as important for characterizing each class (pancreatic cancer, normal pancreatic glands, and normal stroma) and their respective statistical weights.

*m/z*	Lasso Weight		
	Cancer	Normal Glands	Normal Stroma
150.7	0.152		
155.2	−0.012		
155.7			0.201
179.2	0.065		
180.7		−0.071	
186.7	0.060		
187.2	0.028		
187.7	0.005		
189.2		0.034	
196.7			0.414
197.7		−0.009	0.028
198.2			0.041
200.2		−0.231	
202.7	0.159		
204.2	0.252		
207.2			0.026
214.7			0.185
215.2	−0.096		0.251
217.2			0.173
233.7		−0.146	0.983
244.7		−0.193	
246.7			0.192
247.2			0.306
250.2			0.028
264.7	0.008		
265.2	0.008		
265.7	0.002		
266.2	0.011		
267.2	0.002		
277.2	−0.069	0.033	
282.2	−0.026		
306.2	0.118		
307.2	0.174		
307.7	0.174		
308.2	0.110		
331.2	0.136		
331.7	0.025		
332.2	0.202		
332.7	0.051	−0.072	
333.2	0.133		
333.7		−0.017	
335.7	0.077		
344.2		−0.112	
346.2	0.213		
347.2		−0.082	
352.7		−0.182	0.063
359.2		−0.180	
359.7		−0.058	
367.7	0.091		
376.7		−0.001	
381.7		−0.112	
382.7		−0.129	
386.2	0.082		
391.2		0.065	
418.7	0.099		
420.7		−0.009	
421.7		−0.279	
428.2	0.010		
441.7	0.287		
448.7		−0.230	
452.2		0.024	
456.7		−0.311	
498.7		−0.023	
499.2	0.241		
499.7	0.074		
503.2	0.313		
503.7	0.049		
506.7			0.138
556.2		0.309	
574.7		−0.316	
578.2	−0.097	0.173	
614.7	0.007	−0.088	
615.2	0.028		
625.7		−0.073	
626.2	0.016		
646.7		−0.161	
648.2		−0.205	
648.7		−0.210	
649.2	0.245	−0.131	
649.7	0.536	−0.038	
650.2	0.224		
650.7	0.012	−0.411	
669.7		−0.036	
710.8		−0.072	
724.8	0.069		
725.8	0.205		
726.8		−0.063	
737.8		−0.210	
738.3	0.067	−0.064	
738.8	0.130	−0.148	
746.8	0.018		
747.3	0.132		
747.8	0.284		
752.8		−0.034	
760.8		−0.110	
775.8	0.057		
778.8	0.000	−0.202	
780.8		−0.001	
788.8	0.030		
792.8		0.231	
796.8	0.038		
797.8	0.143		
871.8		−0.139	
872.3		−0.121	
872.8		−0.125	
885.8	0.004		
887.8	0.044		
892.8		−0.068	
896.8		−0.025	
904.8		−0.049	
906.8	0.223	−0.223	
907.8		−0.034	

The Lasso yields a classifier that predicts whether a pixel belongs to a certain class based on the highest probability assigned of being pancreatic cancer, normal pancreatic glands, or normal stroma. To test our model using the training set of samples, we performed a 25-fold leave-one-patient-out CV and evaluated the agreement between the prediction obtained by Lasso and the diagnosis obtained by histopathologic evaluation of the same tissue section, which was H&E stained after being imaged by DESI-MSI [26]. An overall agreement of 98.1% was achieved when the total of 45,273 pixels were analyzed in CV. Table 2 shows the results obtained for each class. Note that the normal pancreatic gland class showed the highest agreement (99.3%) with pathologic evaluation, followed by the cancer class (96.4%) and the stroma class (79.9%). There are two key observations that could account for the lower agreement obtained for the stroma class compared to the other classes. First, only 1,389 pixels in our classifier corresponded to pure stroma pixels, a number significantly lower than what was obtained for the other two classes (34,014 pixels of normal pancreatic glands and 9,870 pixels of pancreatic cancer). Second, stromal tissue contained the least amount of detectable ions in the mass spectra, as observed in Fig 2C and, consequentially, the lowest number of statistically significant features was selected for characterizing this class (14 *m/z* values). Nevertheless, when normal pancreatic glands and normal stroma were combined into one class of normal pancreatic tissue, the overall agreement rate increased to 98.7% (Table 3). When we applied our classification model to a set of 17 independent samples with regions of clear histopathologic diagnosis, an overall agreement rate of 98.6% was achieved for the 31,235 pixels considered. The results obtained for each tissue class in the validation set are shown in Table 2. Note that, similarly to the training set of samples, the lowest agreement was observed for the stroma class (83.8%), compared to the agreement obtained for the normal pancreatic gland (99.8%) and cancer (95.4%) classes. When normal stroma and normal pancreatic glands were combined into one class, a high overall agreement of 98.9% was achieved for the independent set of validation samples (Table 3).

**Table 2:** Prediction results for the 76,508 pixels analyzed in the training and validation set of samples, in comparison with pathologic analysis.

Pathology	Predicted			Standard Error	Agreement (Percent)	Overall Agreement (Percent)
	Cancer	Glands	Stroma			
**Training set (45,273 pixels)**						98.1 (0.06 standard error)
Cancer	9,514	148	208	0.19	96.4	
Glands	207	33,787	20	0.05	99.3	
Stroma	31	248	1,110	1.08	79.9	
**Validation set (31,235 pixels)**						98.6 (0.07 standard error)
Cancer	6,448	284	25	0.25	95.4	
Glands	23	23,814	17	0.03	99.8	
Stroma	3	98	523	1.47	83.8	

**Table 3:** Prediction results for the 76,508 pixels analyzed in the training and validation set of samples, in comparison with pathologic analysis, with normal stroma and normal pancreatic glands combined into one class.

Pathology	Predicted		Standard Error	Agreement (Percent)	Overall Agreement (Percent)
	Cancer	Normal			
**Training set (45,273 pixels)**					98.6 (0.06 standard error)
Cancer	9,514	356	0.19	96.4	
Normal	238	35,165	0.04	99.3	
**Validation set (31,235 pixels)**					98.9 (0.06 standard error)
Cancer	6,448	309	0.25	95.4	
Normal	26	24,452	0.02	99.9	

**Fig 4:**
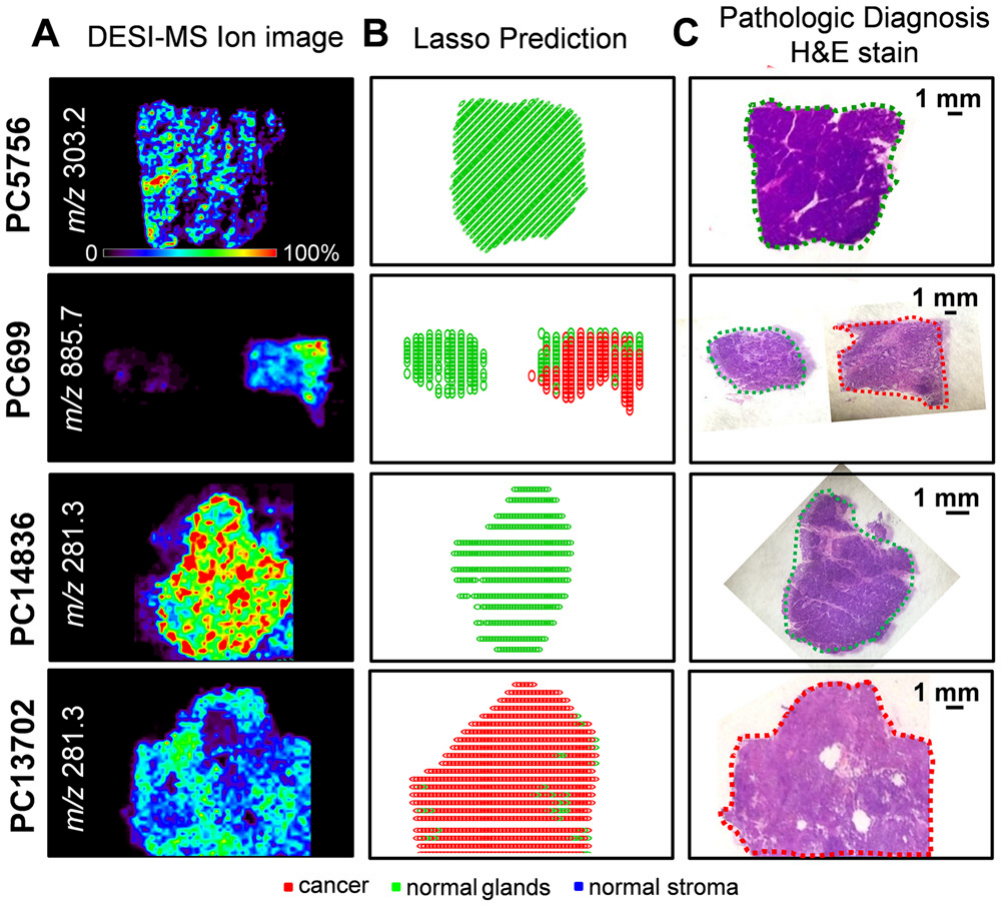
Selected DESI-MSI ion image and Lasso prediction results obtained for training samples PC5756 and PC699 and validation samples PC14836 and PC13702. (A) Negative ion mode DESI-MSI ion images of *m/z* 303.2, *m/z* 885.7, *m/z* 281.3, and *m/z* 281.3 are shown for samples PC5756, PC699 (pair of normal pancreas and pancreatic cancer), PC14836, and PC13702, respectively. (B) Lasso prediction results are shown for each sample, with pixels predicted as pancreatic cancer shown in red, normal pancreatic glands in green, and normal stroma in blue. (C) Optical images of the H&E-stained tissue sections are shown, with the regions diagnosed by the pathologist delineated using the same color representation.

To spatially observe the results of our classification system, two-dimensional false-color images were plotted showing the results for cancer as red pixels, normal pancreatic glands as green pixels, and normal stroma as blue pixels. These images can be directly compared to the optical images of the H&E-stained tissues. Fig 4 shows the CV classification results obtained for samples PC5756 and PC699 from the training set and for samples PC14836 and PC13702 from the validation set. Note that discrepancies in the shapes of the Lasso predicted images and the DESI-MSI images occurred for some examples due to (1) the segmentation algorithm used to select regions for statistical analysis, (2) the exclusion of pixels near the boundaries of the tissue section where background ions from glass slide (which do not contribute to the molecular information) are observed at high intensities, and (3) the suboptimal aspect ratio of the predicted Lasso images, which required rescaling and adjusting. Optical images of the H&E-stained sections with pathologic diagnosis for these samples are shown in Fig 4. As observed, the discrepancies observed between statistical results and histopathologic evaluation occur for a low number of pixels within each sample. As our approach is performed and evaluated on a pixel by pixel basis, this is not surprising: regions with a predominant histologic feature may contain few cells of a different histologic class.

### Statistical Predictions for Mixed Samples

**Fig 5:**
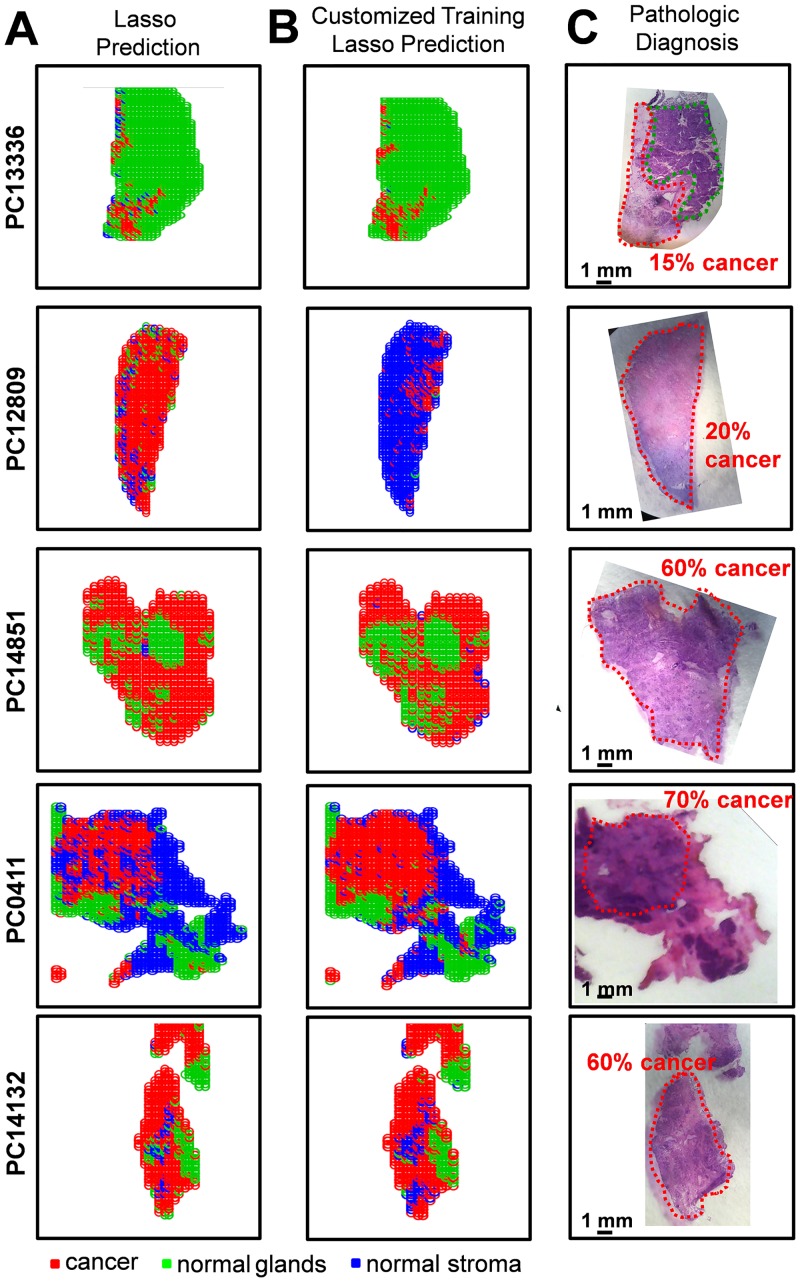
Statistical predictions in comparison with histopathologic diagnosis for samples of mixed histology. Results are shown for samples PC13336, PC12809, PC14851, PC0411, and PC14132 using (A) the Lasso classification system built with the training set of samples and (B) the customized training set approach. Pixels predicted as pancreatic cancer are shown in red, normal pancreatic glands in green, and normal pancreatic stroma in blue. (C) Optical images of the H&E-stained tissue sections are shown, with the regions diagnosed by the pathologist delineated using the same color representation.

A large number of the samples analyzed in our study contained regions of high cellular heterogeneity, with a mixed composition of cancer cells infiltrating normal pancreatic glands and normal stromal cells. Careful histologic evaluation was performed in order to assign and accurately describe these regions with mixed histologic features. Regions of cellular composition not accounted for when building our statistical models (such as lymphocytes, acute inflammation, and necrosis) were excluded from our statistical prediction approach. To assist with the evaluation of the DESI-MSI/Lasso results, a visual measurement of the percent cell composition was given by the pathologist for selected regions of the tissue section. For example, sample PC13336 contained a region of pure normal pancreatic glands, adjacent to a region of 15% tumor cells and 85% normal pancreatic gland cells. Sample PC12809 presented a mixed composition of 20% tumor cells within 80% stromal cells. Sample PC14851 presented 60% tumor cells mixed within 40% normal pancreatic glands. Sample PC0411 presented a region with high tumor cell composition (70%) mixed with normal stroma (30%), surrounded by normal pancreatic glands and stroma. Sample PC14132 presented a region with 60% tumor cells intermingled with normal stroma, while the remaining tissue was composed of both normal pancreatic glands and normal stromal tissues. Optical images of the H&E-stained tissue sections delineated by histopathologic evaluation are shown in Fig 5.

Due to the complex nature of these samples, the results for each sample were evaluated separately in order to assess the performance of our approach. The agreement between the DESI-MSI/Lasso prediction and the diagnosis given by histopathology was evaluated both in terms of the spatial distribution of pixels and the percent tumor/normal cell composition for the specific tissue region. For example, an excellent agreement was observed for sample PC13336, with the region of normal pancreatic glands completely assigned as normal glands, and the adjacent region of 15% tumor cell concentration and 85% normal pancreatic gland concentration assigned by the statistical approach as 12% cancer pixels within mostly normal pancreatic glands (Fig 5). Another sample in which excellent tissue composition and spatial agreement was observed was PC0411. The region with high tumor cell concentration (70%) mixed with stroma (30%) as diagnosed by histopathology was correctly classified by our approach, as well as the surrounding region of normal pancreatic glands and stroma. On the other hand, sample PC14132 was classified as containing a higher tumor cell composition than that assessed by histopathology, with regions diagnosed as normal glands assigned as containing cancer. Overall, results obtained with the developed Lasso approach showed outstanding agreement with histopathology for both cell concentration and spatial distribution for 70% of the samples evaluated. For the remaining 30% of samples, the disagreement was caused by either an overprediction of cancer cell composition (20% of samples) or false-negative prediction (5% samples). Results for the samples described above are shown in Fig 5A.

An alternative approach commonly used to evaluate results from complex samples is the “majority rule” approach, where an overall agreement is given for an entire sample based on the majority of the pixels assigned (i.e., if >50% pixels are predicted as cancer, the sample is predicted as “cancer”). Using the majority rule approach, 95% agreement was achieved for the mixed samples using our classification system. Nevertheless, because the majority rule approach disregards important spatial features of the samples, which are crucial for surgical margin evaluation, we have chosen not to evaluate our results by this method. Instead, in an effort to optimize our methods for mixed samples, we applied a novel customized training statistical method using the Lasso recently developed for MS data [29]. This customized training strategy makes predictions on the test dataset when the features of the test data are available at the time of model fitting. The data are clustered to find training points close to each test point, and then the method fits a Lasso model separately in each training cluster. This means that a customized training set is generated from the data in the training set for each test sample. The method also generates a separate, unique list of statistically significant features (*m/z* values) for each test sample. This procedure, by incorporating an entire dataset of training samples, is useful in situations where complex datasets have underlying structure that could account for difficulties in prediction. When using the customized training approach, our agreement increased to 81% of all mixed samples analyzed. Increased agreement was mostly observed for samples that had previously been classified by our approach as having a higher tumor cell composition than what was assessed by histopathology, with many “false positive” samples now correctly classified as only normal tissue. For example, sample PC12809, a mixed composition of 20% tumor cells within 80% stromal cells, was classified as being composed of 81% cancer by our traditional Lasso approach. Using the customized training set approach, 15% of the pixels were classified as cancer and the remaining 85% as normal stroma, a much better agreement. Results for the samples discussed above using this new customized approach are shown in Fig 5B.

### Surgical Margin Evaluation

**Fig 6:**
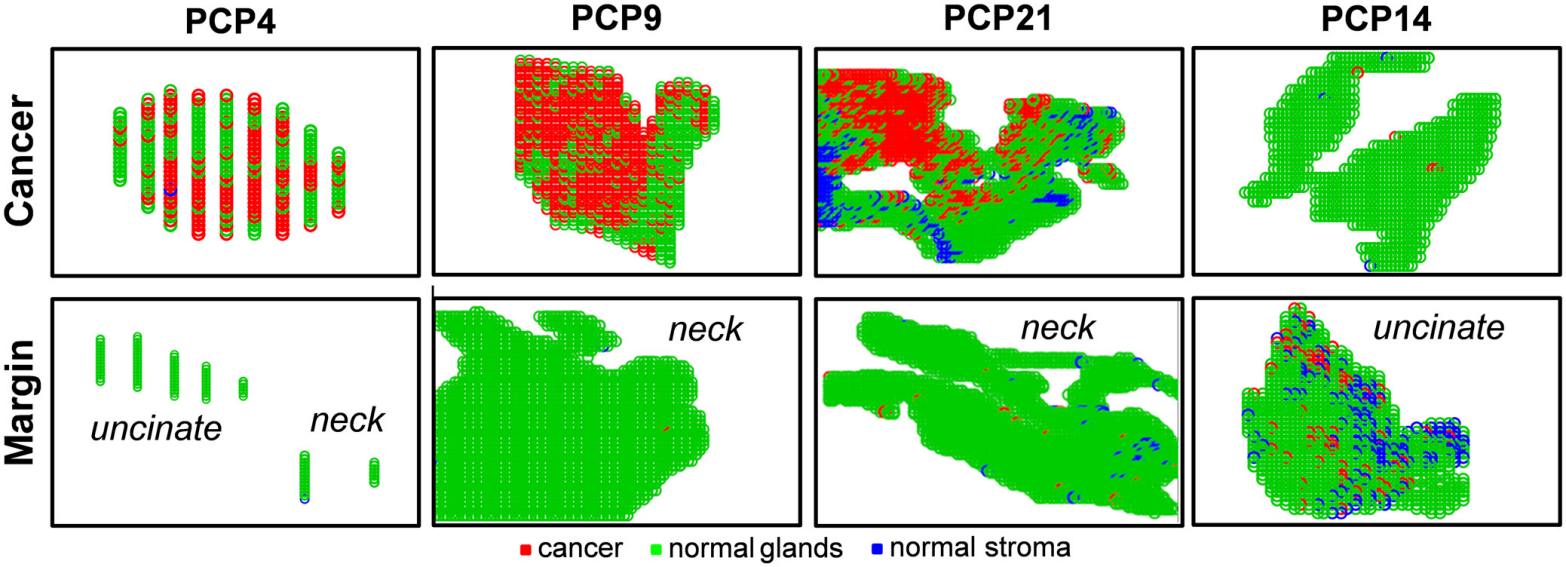
Lasso prediction results for cancer and margin tissues obtained prospectively from surgery in pancreatic cancer patients. Lasso prediction results are shown for the cancer tissue as well as the surgical margins (uncinate and/or neck) of surgical cases PCP4 (negative margins), PCP9 (negative margin), PCP21 (negative margin), and PCP14 (positive margin). Pixels predicted as pancreatic cancer are shown in red, normal pancreatic glands in green, and normal stroma in blue.

To evaluate the results for the surgical cases in our study, we used the customized training set approach described above. Thirty-two pancreatic cancer patients were recruited for our study, and a total of sixty-five samples were collected (Table 4). In most cases, serial sections of at least one of the margins (neck and/or uncinate) were obtained, as well as a section of the tumor. The results from DESI-MSI/Lasso analysis were not fed back to the surgeons during the procedure, but independently evaluated post-operatively. For surgical case PCP4, for example, a sample of the cancerous tissue as well as sections of the neck and uncinate margins were obtained during surgical resection and analyzed by DESI-MSI. Histologic evaluation of the surgical margins both intraoperatively and after DESI-MSI diagnosed both margins as negative for the presence of cancer. The uncinate margin was composed of 90% normal pancreatic glands and 10% stroma, while the neck margin was composed of normal pancreatic glands only. The tumor section contained 60% tumor cells, with the remaining being composed of normal pancreatic glands and stroma. Predictions were performed using the customized training set approach, and results are shown in Fig 6. As observed, a good agreement between histopathologic evaluation and our analysis was achieved. In particular, both margins were detected as being purely normal, with stroma pixels detected by our classifier for the neck margin, whereas 55% of pixels were detected as cancerous for the cancer section, as described by histopathology. Similar results were obtained for the majority of cases with negative margins, including PCP9 (Fig 6) and PCP31.

**Table 4:** Clinicopathologic characteristics of 32 patients who underwent resection of pancreatic cancer and were prospectively evaluated.

Characteristic	Number (Percent)
**Age (years)**	69 (46–85)*
**Male gender**	16 (50%)
**Type of operation**	
Whipple pancreaticoduodenectomy	26 (81%)
Distal pancreatectomy	6 (19%)
**T stage**	
T3	25 (78%)
T2	6 (19%)
T1	1 (3%)
**Node positive**	20 (62%)
**Perineural invasion**	22 (69%)

*Median (range).

For case PCP21, samples of tumor and neck margin were obtained. While the neck margin was diagnosed as negative by histopathology (95% normal pancreatic glands and 5% normal stroma), the tumor section contained a region with 80% tumor cell concentration within stroma cells, and the remaining tissue was diagnosed as tumor infiltrating normal glands and stroma, with a low tumor cell concentration. Results obtained by our approach are shown in Fig 6. While high spatial and tumor/normal cell composition agreement was obtained for the cancerous tissue, a very small number of pixels (2%) within the normal neck margin were detected as being cancerous by our method. Note that this error is within the error rates we obtained when developing the statistical approach. Similar results were observed for other large margin samples (over 1,000 pixels) evaluated by our classifier, including the negative neck margins from surgical cases PCP20 and PCP28. Yet an excellent agreement was observed for the cancerous tissues for both surgical cases PCP20 and PCP28.

**Fig 7:**
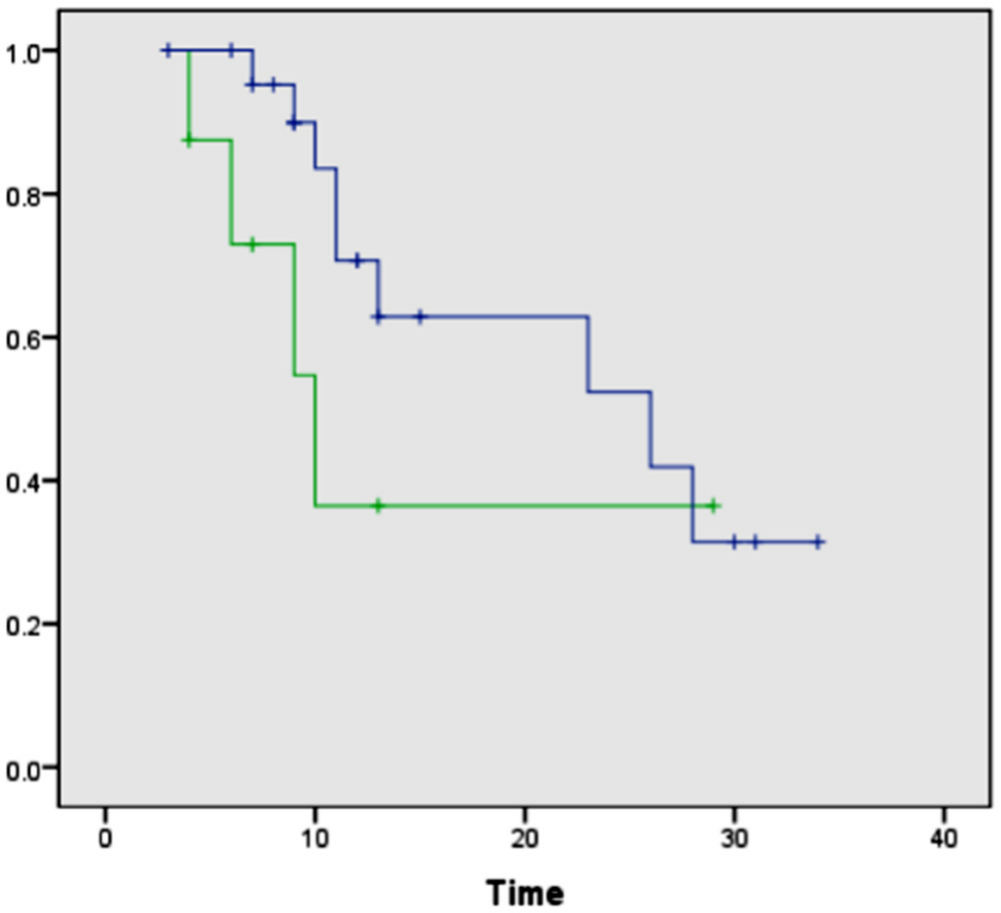
Kaplan-Meier curves comparing overall survival after surgical resection between patients with negative surgical margins by both DESI-MSI/Lasso and frozen section analysis and patients with positive margins by DESI-MSI/Lasso but negative margins by frozen section analysis. The blue line indicates patients with negative surgical margins by both DESI-MSI/Lasso and frozen section analysis (*n =* 23, median 26 mo); the green line indicates patients with positive margins by DESI-MSI/Lasso but negative margins by frozen section analysis (*n =* 8, median 10 mo). Log-rank test, *p* = 0.209. One patient in the prospective arm of the study had positive margins by both techniques and was excluded from this comparison.

PCP14 was the only surgical case for which a positive margin was found intraoperatively for the uncinate margin. Histologic evaluation of the serial tissue section analyzed in the laboratory by DESI-MSI also detected the presence of tumor cells within the uncinate margin, about 10% tumor cell concentration infiltrating within normal glands and normal stroma. This positive margin was also detected by our DESI-MSI/Lasso approach, with an excellent agreement of 12% pixels detected as cancer among normal glands and stroma. Overall, good agreement between histopathologic evaluation and the DESI-MSI/Lasso results was obtained in our study for 24 of the surgical cases evaluated. In four of the remaining surgical cases, a false positive was observed in surgical margins, with as many as 20% of the pixels classified as cancer by our approach while diagnosed as normal by histopathology. In the other four cases, a maximum of 2% of pixels were classified as cancerous in surgical margin tissue while diagnosed as normal by histopathology. It was interesting to note that the median survival after resection for these eight patients with false-positive margins by DESI-MSI/Lasso but not by histopathologic examination was only 10 mo, as opposed to 26 mo for patients with negative margins by both DESI-MSI/Lasso and histopathology (Fig 7). Historically, a median survival of 10 mo is what one would expect from a margin-positive pancreatic cancer resection and 26 mo from a margin-negative resection. Although this difference did not reach statistical significance (*p* = 0.209), likely due to small sample size, it would be intriguing to hypothesize that our method was more “sensitive” than frozen section analysis (the standard method currently) in detecting margin involvement by tumor in patients who experienced early recurrence and death. Further study is warranted to prove or disprove the aforementioned hypothesis. False-negative results were not observed by our approach when evaluating surgical margins. Excellent agreement was observed for all the cancer tissues analyzed from surgical cases.

## Discussion

Accurate intraoperative evaluation of resection margins in pancreatic cancer (as with any oncologic) surgery is critical to overall surgical success and patient survival. In this study, we used DESI-MSI and the Lasso method to develop an automated system to classify pancreatic tissue sections based on molecular information. In total, 254,235 individual mass spectra were considered in our approach, and classified as normal pancreatic glands, normal pancreatic stromal tissue, or pancreatic cancer. DESI-MSI molecular profiles obtained for the samples showed high relative abundances of many ions identified as FAs and GPs. The method was developed and tested using different sets of training and validation samples, and its performance was evaluated on a per pixel basis in comparison to histopathologic diagnosis. Using a set of independent validation samples with unequivocal histologic features, classification results were in agreement with pathologic diagnosis in 98.6% of the pixels evaluated. The highest error value in classification was observed in the normal stroma class, which presented low abundances of molecular species by DESI-MSI compared to the other tissue classes, and for which the lowest number of samples/pixels was obtained. A set of complex validation samples with mixed histologic features was carefully evaluated using a statistical approach that employs Lasso to generate a customized training set for each test sample considered. The results obtained were methodically compared with the histopathologic results for both spatial features and cellular composition in each sample. When judged by both criteria, 81% agreement was obtained. This is the first study to our knowledge to report the use of ambient ionization MS imaging for the diagnosis of pancreatic cancer.

We further demonstrated the value of DESI-MSI/Lasso for surgical margin evaluation in pancreatic cancer surgery using neck and/or uncinate margins and tumor tissues prospectively collected from 32 surgical procedures performed at Stanford University Hospital. Samples were imaged and classified using the customized training set approach developed for mixed samples. Using our method, we were able to correctly diagnose cancer in a case where a positive neck margin was observed by histopathology. For the remaining cases, all margins were diagnosed as negative by histopathologic analysis. In nine of the 32 cases, our method classified pixels (1%–20%) in the neck and/or uncinate margin as cancerous while histopathology did not. This disagreement in diagnosis could be attributed to the inherent error range in our analysis (~2%), especially for margin samples that are large and contain over 1,000 pixels. However, the early recurrence and death noted in patients with false-positive margins by DESI-MSI raises the question as to whether these margins were truly positive, and accurately classified by our method but not by the histopathologic analysis of frozen sections.

The classification results obtained using Lasso are similar to what has been reported for other cancers (>90% accuracy in CV) [13]. Improvements in our classification system will be sought by increasing sample size for stroma tissue, which contributes to most of the confusion in our classification system. As DESI-MSI is performed in the ambient environment with minimal sample preparation requirements, we believe this technology is attractive for routine use in clinical practice. Furthermore, as DESI-MSI evaluation is performed in real time, it is typically faster than frozen section analysis. Note, however, that in this study DESI-MSI was performed on the entire tissue section in order to unambiguously correlate and compare the molecular results with histopathologic diagnosis. In some cases, over 2 h were necessary to image a large surgical margin tissue. Thus, the timeframe involved in imaging analysis by DESI-MSI could become a limitation for its routine use in the clinic. However, we expect that in clinical practice, DESI-MSI analysis would be rapidly performed in selected regions of the tissue that present diagnostic ambiguity (<1 s/pixel), in a profiling and not imaging mode. In this way, DESI-MSI would serve as a rapid adjunct technique to the standard method of frozen section analysis, and thereby enhance intraoperative margin assessment by the pathologist.

Further limitations of incorporating DESI-MSI into clinical practice include the required staffing resources and cost. Currently, data recording, interpretation, and statistical analysis are performed after sample analysis, which requires time and expertise. Improvements in computational methods for data acquisition and processing are necessary to successfully translate the technology to clinical practice. In addition, current commercially available mass spectrometers are costly instruments (>$200,000) that require regular maintenance. Yet, as many efforts are underway to develop smaller and cheaper mass spectrometers, we expect these instruments to become more accessible to hospitals for clinical use [32,33].

In summary, this study demonstrates that DESI-MSI/Lasso can be successfully used to classify tissue as normal or pancreatic cancer. Our findings provide evidence that the molecular information obtained by DESI-MSI/Lasso from tissue samples has the potential to transform the evaluation of surgical specimens. With further development and automation, we believe the described methodology could be routinely used for surgical margin assessment of pancreatic cancer. Yet, careful evaluation of the long-term benefits to patients of the use of DESI-MSI for surgical margin evaluation is needed, using a larger cohort of cases, to determine its proper value in clinical practice.

## Supporting Information

S1 Text STROBE statement. (DOCX)

## Abbreviations

CV: cross-validation
DESI-MSI: desorption electrospray ionization mass spectrometry imaging
FA: fatty acid
GP: glycerophospholipid
IRB: institutional review board
Lasso: least absolute shrinkage and selection operator
MS: mass spectrometry
PC: glycerophosphocholine
PG: glycerophosphoglycerol
PI: glycerophosphoinositol
PS: glycerophosphoserine
