# Supplementary material for: Pancreatic Cancer Surgical Resection Margins: Molecular Assessment by Mass Spectrometry Imaging
Source: PLoS Med. 2016 Aug 30;13(8):e1002108. doi: 10.1371/journal.pmed.1002108 (PMC5019340; doi:10.1371/journal.pmed.1002108)
Supplement: S1 Text — (DOCX) [file pmed.1002108.s001.docx]

STROBE Statement—checklist of items that should be included in reports of observational studies

|  | **Item**  **No** | **Recommendation** |
| --- | --- | --- |
| **Title and abstract** | 1 | (*a*) Indicate the study’s design with a commonly used term in the title or the abstract: Described in the title page of the manuscript. |
|  |  | (*b*) Provide in the abstract an informative and balanced summary of what was done  and what was found: Described in the “Abstract” of the manuscript. |
| **Introduction** |  |  |
| Background/rationale | 2 | Explain the scientific background and rationale for the investigation being reported: Described in the “Introduction” of the manuscript. |
| Objectives | 3 | State specific objectives, including any prespecified hypotheses: Described in the last paragraph of the “Introduction” of the manuscript. |
| **Methods** |  |  |
| Study design | 4 | Present key elements of study design early in the paper: Described in the “Materials and Methods” sections of the manuscript. |
| Setting | 5 | Describe the setting, locations, and relevant dates, including periods of recruitment,  exposure, follow-up, and data collection: Described in the “Materials and Methods” of the manuscript. |
| Participants | 6 | (*a*) *Cohort study*—Give the eligibility criteria, and the sources and methods of selection of participants. Describe methods of follow-up: Described in the “Materials and Methods - Banked Human Malignant and Benign Pancreatic Tissues” of the manuscript and in “Materials and Methods - Prospective Collection of Surgical Samples” of the manuscript.  *Case-control study*—Give the eligibility criteria, and the sources and methods of case ascertainment and control selection. Give the rationale for the choice of cases and controls  *Cross-sectional study*—Give the eligibility criteria, and the sources and methods of  selection of participants |
|  |  | (*b*) *Cohort study*—For matched studies, give matching criteria and number of exposed and unexposed  *Case-control study*—For matched studies, give matching criteria and the number of  controls per case |
| Variables | 7 | Clearly define all outcomes, exposures, predictors, potential confounders, and effect  modifiers. Give diagnostic criteria, if applicable: Described throughout the “Results” and the “Discussion” of the manuscript. |
| Data sources/  measurement | 8* | For each variable of interest, give sources of data and details of methods of assessment (measurement). Describe comparability of assessment methods if there  is more than one group: Described throughout the “Results” and the “Discussion” of the manuscript. |
| Bias | 9 | Describe any efforts to address potential sources of bias: Described in the “Materials and Methods – Mass Spectrometry Imaging” of the manuscript. |
| Study size | 10 | Explain how the study size was arrived at: We took a sufficient number of samples to allow a meaningful statistical analysis. |
| Quantitative variables | 11 | Explain how quantitative variables were handled in the analyses. If applicable,  describe which groupings were chosen and why: Presented in “Results” section.  R“Results” sec |
| Statistical methods | 12 | (*a*) Describe all statistical methods, including those used to control for confounding: Described in the “Materials and Methods – Statistical Analysis” of the manuscript. |
|  |  | (*b*) Describe any methods used to examine subgroups and interactions: Not applicable to this study. |
|  |  | (*c*) Explain how missing data were addressed: No data was missing. |
|  |  | (*d*) *Cohort study*—If applicable, explain how loss to follow-up was addressed: Not applicable to this study.  *Case-control study*—If applicable, explain how matching of cases and controls was addressed: Not applicable to this study.  *Cross-sectional study*—If applicable, describe analytical methods taking account of  sampling strategy: Not applicable to this study. |
|  |  | (*e*) Describe any sensitivity analyses: We performed cross-validation as described in the |
| Continued on next page |  | “Materials and Methods –Statistical Analysis” of manuscript. |
|  |  |  |

| **Results** |  | |
| --- | --- | --- |
| Participants | 13* | (a) Report numbers of individuals at each stage of study—eg numbers potentially eligible, examined for eligibility, confirmed eligible, included in the study, completing follow-up, and analysed: Described in the “Materials and Methods - Banked Human Malignant and Benign Pancreatic Tissues” of the manuscript and in “Materials and Methods - Prospective Collection of Surgical Samples” of the manuscript. |
|  |  | (b) Give reasons for non-participation at each stage: No patient refused to participate in the prospective aspect of this study. 32 patients were approached for consent and 32 consented. |
|  |  | (c) Consider use of a flow diagram: A flowchart of the study is provided as Figure 1. |
| Descriptive  data | 14* | (a) Give characteristics of study participants (eg demographic, clinical, social) and information  on exposures and potential confounders: Clinicopathologic characteristics of 32 patients recruited are provided in Table 3.  cancer and were orospectively evaluated. |
|  |  | (b) Indicate number of participants with missing data for each variable of interest: Not applicable to this study. |
|  |  | (c) *Cohort study*—Summarise follow-up time (eg, average and total amount): Not applicable to this study. |
| Outcome data | 15* | *Cohort study*—Report numbers of outcome events or summary measures over time: See Fig. 7. |
|  |  | *Case-control study—*Report numbers in each exposure category, or summary measures of  exposure |
|  |  | *Cross-sectional study—*Report numbers of outcome events or summary measures |
| Main results | 16 | (*a*) Give unadjusted estimates and, if applicable, confounder-adjusted estimates and their precision (eg, 95% confidence interval). Make clear which confounders were adjusted for and  why they were included: Described throughout the “Results” and the “Discussion” of the manuscript. |
|  |  | (*b*) Report category boundaries when continuous variables were categorized: Described throughout the “Results” and the “Discussion” of the manuscript. |
|  |  | (*c*) If relevant, consider translating estimates of relative risk into absolute risk for a meaningful  time period: Not relevant. |
| Other analyses | 17 | Report other analyses done—eg analyses of subgroups and interactions, and sensitivity  Analyses: Described throughout the “Results” and the “Discussion” of the manuscript. |
| **Discussion** |  |  |
| Key results | 18 | Summarise key results with reference to study objectives: Described in the first and second paragraphs of the “Discussion” of the manuscript. |
| Limitations | 19 | Discuss limitations of the study, taking into account sources of potential bias or imprecision.  Discuss both direction and magnitude of any potential bias: Described in the third and fourth paragraphs of the “Discussion” of the manuscript. |
| Interpretation | 20 | Give a cautious overall interpretation of results considering objectives, limitations, multiplicity  of analyses, results from similar studies, and other relevant evidence: Presented in the last paragraph of the “Discussion” of the manuscript. |
| Generalisability | 21 | Discuss the generalisability (external validity) of the study results: Presented in the last paragraph of the manuscript. |

**Other information**

Funding 22 Give the source of funding and the role of the funders for the present study and, if applicable, for the original study on which the present article is based: Provided in the manuscript.

*Give information separately for cases and controls in case-control studies and, if applicable, for exposed and unexposed groups in cohort and cross-sectional studies.

**Note:** An Explanation and Elaboration article discusses each checklist item and gives methodological background and published examples of transparent reporting. The STROBE checklist is best used in conjunction with this article (freely available on the Web sites of PLoS Med[icine at http://www.plosmedicine.org/,](http://www.plosmedicine.org/) Annals of Internal Medicine at [http://www.annals.org/,](http://www.annals.org/) and Epidemiology [at http://www.epidem.com/).](http://www.epidem.com/) Information on the STROBE Initiative is available at [www.strobe-statement.org.](http://www.strobe-statement.org)
